# Health Information Source Characteristics Matter: Adapting the Dissemination of an HPV Vaccine Intervention to Reach Latina and Vietnamese Women

**DOI:** 10.1007/s10935-021-00643-2

**Published:** 2021-07-20

**Authors:** Suellen Hopfer, Huong T. Duong, Samantha Garcia, Sora P. Tanjasiri

**Affiliations:** 1 Department of Health, Society & Behavior, UCI Chao Family Comprehensive Cancer Center, University of California, Irvine, CA USA; 2 Anteater Instruction & Research Building (AIRB) Second Floor, Office 2024, 653 E. Peltason, Irvine, CA 92697 USA; 3 Department of Epidemiology, University of California, Irvine, CA USA; 4 Director of Community Engagement, UCI Chao Family Comprehensive Cancer Center, Orange, CA USA

**Keywords:** Communication channel use, Channel Complementarity Theory, Health communication, Health information seeking, HPV vaccination, Latina women, Vietnamese women, Intervention implementation, Adaptation, Dissemination

## Abstract

Latina and Vietnamese women are disproportionately burdened by human papillomavirus (HPV)-associated cervical cancer and underutilize the HPV vaccine, which is an effective cancer prevention measure. To inform the adaptation of a National Cancer Institute’s evidence-based cancer control program, HPV Vaccine Decision Narratives, and because of the rapidly changing information concerning consumption patterns of young adults, we elicited preferences and characteristics associated with women’s interest in various health information sources, specifically for HPV vaccination. We conducted 50 interviews with young Latina and Vietnamese women at two Planned Parenthood health centers in Southern California. Interview questions were guided by the Channel Complementarity Theory and focused on understanding the multiple communication channels women turn to for health, and the characteristics that motivate their use. Our results showed that Latina and Vietnamese women turn to many sources, from online and social media to school health classes, mothers, and doctors. Specific characteristics that motivate women’s online use of health information included immediacy and access, convenience and credibility. When receiving HPV vaccine information, privacy, avoiding information overload, interpersonal engagement, and receiving health information from trusted sources was important. Our study advances the prevention literature by elevating the role of platform delivery considerations and emphasizing preferences to effectively reach Latina and Vietnamese women, who are disproportionately burdened by HPV cancers and are less aware of HPV vaccine prevention. These results can be used to further inform the dissemination of this cancer control program. Adaptation of the program should include changes to its delivery, such that trusted sources are used, and dissemination is coordinated to send a consistent message across multiple communication methods.

## Introduction

Latina and Vietnamese women in America are disproportionately burdened with cervical cancer associated with the human papillomavirus (HPV). At 10.2 per 100,000, Latina women experience the highest cervical cancer incidence rate of any racial or ethnic group (Siegel et al., 2016); Vietnamese women experience a five-fold risk relative to White women at 7 per 100,000 (Jin et al., 2016; Mann et al., 2015; National Cancer Institute [NCI], 2017). Although research on subgroups of Asian American women remains limited and their risk relative to other races and Asian ethnicities remains unexplored (Chen et al., 2018), some studies have shown that Vietnamese women have higher cervical cancer rates than other Asian American ethnicities, including Chinese, Filipinas, and Koreans (American Cancer Society, 2016; Gomez et al., 2013; Telli et al., 2011; Wang et al., 2010).

At the same time, minority young adult women underutilize the HPV vaccine, which can prevent HPV-related cancers. In 2017, 38.8% of Latinas and 45.2% of Asian Americans aged 19–26 reported initiating the HPV vaccine, which requires three doses (Hung et al., 2019). To date, only community-based studies in the United States have explored HPV vaccine initiation among Vietnamese women (Yi et al., 2013). Healthy People 2030 recommends the reduction of HPV infections among young adults aged 20–34 through increased HPV vaccination (U.S. Department of Health and Human Services, 2020). Following the U.S. Food and Drug Administration’s licensing of the HPV vaccine for use until age 45, the Centers for Disease Control and Prevention’s Advisory Committee on Immunization Practices (CDC ACIP) now recommends HPV vaccination for people until the age of 26 and makes the vaccination available to women until age 45 (Meites et al., 2019).

We conducted in-depth interviews with two unique populations in Southern California composed of Latina and Vietnamese women visiting Planned Parenthoods in Orange County (known as Planned Parenthood of Orange and San Bernardino Counties, or PPOSBC, 2019). We investigated where women turned for health information and their preferences for receiving HPV vaccine information (with the aim to adapt the dissemination of an existing evidence-based cancer control program [EBCCP] called HPV Vaccine Decision Narratives). California’s Orange County is 34% Latino and 21% Asian American, including the largest population of Vietnamese in the United States (City of Santa Ana, 2021; U.S. Census Bureau, 2019). This county is known for its Vietnamese ethnic enclave and includes the city of Westminster, which is dubbed “Little Saigon.” Vietnamese Americans in Orange County constitute the highest concentration (39%) of all Asian Americans in the country (U.S. Census Bureau, 2019). Additionally, the city of Santa Ana in Orange County is known for its Latino cultural heritage and majority Latino population (78%; U.S. Census Bureau, 2019).

We partnered with PPOSBC to reach medically underserved young adult Latina and Vietnamese women, who have high rates of new cervical cancer cases coinciding with low rates of HPV vaccination completion and high population prevalence in Orange County. Planned Parenthood is the leading U.S. provider of women’s sexual and reproductive health care (PPOSBC, 2019). Women attending PPOSBC health centers are likely to come from communities with relatively low socioeconomic status and are unlikely to receive routine preventive care (PPOSBC, 2019). An important step in addressing cancer health disparities involves more effectively reaching these minority populations and more fully appreciating the sources to which Latina and Vietnamese turn for trusted health information.

We explored Latina and Vietnamese women’s general health information-seeking behaviors and their preferences for receiving HPV vaccine information, given young adults’ rapidly changing (health) media consumption patterns. We also intended this study to inform the adaptation and dissemination of a National Cancer Institute (NCI) EBCCP called HPV Vaccine Decision Narratives to more effectively reach Latina and Vietnamese women with vaccine messages and, thus, to reduce the incidence of HPV-related cancer in this population (NCI, 2015).

HPV Vaccine Decision Narratives is a series of brief videos presenting young adult women’s HPV vaccine decision stories interwoven with relevant statistics about HPV-related cancers (NCI, 2015). The intervention was originally developed to reach college women, and in the context of a randomized controlled trial the decision story messages nearly doubled vaccinations in the intervention relative to the control group (Hopfer, 2012). The EBCCP has since been adapted to reach many audiences, including women who seek care at safety-net clinics like Planned Parenthood (Hecht et al., 2021; Hopfer, Garcia, et al., 2017a; Hopfer Ray et al., 2017b).

Given rapidly changing media environment and health information consumption trends, identifying a target audience’s preferences for where and from whom they seek and receive health information is critical for effectively adapting an EBCCP. As part of a larger study, we sought to identify Latina and Vietnamese women’s preferences for where and from whom they seek and receive health information to adapt the HPV vaccine NCI EBCCP to effectively reach women seeking care at Planned Parenthood health centers.

Young adults’ media consumption patterns continue to rapidly expand (Pew Research Center, 2019). Asian Americans have shown higher rates of smartphone ownership (~ 82%) than their non-Latino White (~ 70%) and Latino (68%) counterparts (Bender et al., 2014; Pew Research Center, 2019). Foreign-born Latinos and non-English native speakers may be less likely to have reliable internet access and, therefore, have lower odds of engaging in online health information-seeking behavior or relying on other channels for health information (Gonzalez et al., 2016). Media trends show an expanding range of communication methods, especially social media and online sources, that may be used to access health information (Anderson, 2019; Kim & Kreps, 2020; Pew Research Center, 2019, 2021). There is a critical need to investigate how and when young adults, especially minority underserved women, seek health information, how they prefer to receive HPV vaccine prevention messages, and what communication mode characteristics they value (Anderson, 2015; Pew Research Center, 2019).

Recognizing the rapidly changing media environment and health-seeking behaviors of young adults, we based our study on the Channel Complementarity Theory (CCT; Dutta-Bergman, 2004b), which is a theoretical framework that offers insight into the motivations behind using many channels to gather health information. We defined communication channels in this study as any communication source that may provide health information (examples include in-person medical experts, the internet, social media platforms, radio, and print). Our focus on health information source or channel preferences takes on new weight in the context of the coronavirus pandemic, when digital online health information seeking has dramatically increased (Liu, 2020). CCT is rooted in the notion that individuals who are motivated to acquire information about a given topic or issue will use all sources that they perceive as meeting their information needs and that will complement rather than displace one another (Dutta-Bergman, 2004a). Ruppel and Rains (2012; also see Rains & Ruppel, 2016) extended the theory to consider the source characteristics of health information. These may include the degree to which a source is convenient, anonymous, tailored, and offers access to medical expertise. In today’s contemporary media environment, young adult minority populations use many sources for health information, including interactive social media sites, health care providers, and print media (Anderson, 2015; Pew Research Center, 2019, 2021). Consequently, it is increasingly important to understand what communication channels are valued and what motivates their use to effectively reach racial and ethnic minority audiences with prevention messages.

## Method

### Participants and Data Collection

In late summer 2016, we recruited a purposive sample of 24 Latina women, 24 Vietnamese American women, and two staff members from PPOSBC health centers. We conducted semi-structured interviews with them. The staff members, one from each of two PPOSBC health centers, were also interviewed to gauge health practitioners’ perspectives on HPV sequelae as observed in clinic, gain an understanding of the HPV conversations taking place between practitioners and patients, and inform strategies for disseminating the EBCCP brief video HPV vaccine intervention to effectively reach women.

Women patients between ages 18 and 24 were recruited from the waiting rooms at the two PPOSBC health centers in Orange County, California, in the cities of Westminster (serving a majority of Vietnamese clients) and Santa Ana (serving a majority of Latina clients). Women were approached by trained interviewers of the same gender, age, and ethnicity who used the same language that the women used at check-in (i.e., English, Spanish, or Vietnamese). We asked women if they would consider completing a 30-min in-person interview in a PPOSBC office regarding HPV vaccination before or after their clinic visit. While we verbally offered to conduct interviews in the language participants used at check-in, all participants preferred that the interview be conducted in English.

All procedures received human subjects research participation approval from the Planned Parenthood Federation of America and the University of California Irvine. We provided a study information sheet before women agreed to be interviewed. A waiver of signed consent was approved by University of California Irvine, because the signature would have been the only identifiable link to the participant. Each participant received $20 as compensation for her time.

### Interview Guide

We developed the interview guide, which included questions focused on women’s general health information-seeking behaviors and their preferences for receiving education about HPV vaccination. Two of the authors are bilingual–bicultural Vietnamese American and bilingual–bicultural Mexican American whose age and race and ethnicity matched our target audience, and they co-developed the interview guide to ensure that questions were culturally appropriate. We audio-recorded all interviews.

**Fig. 1:**
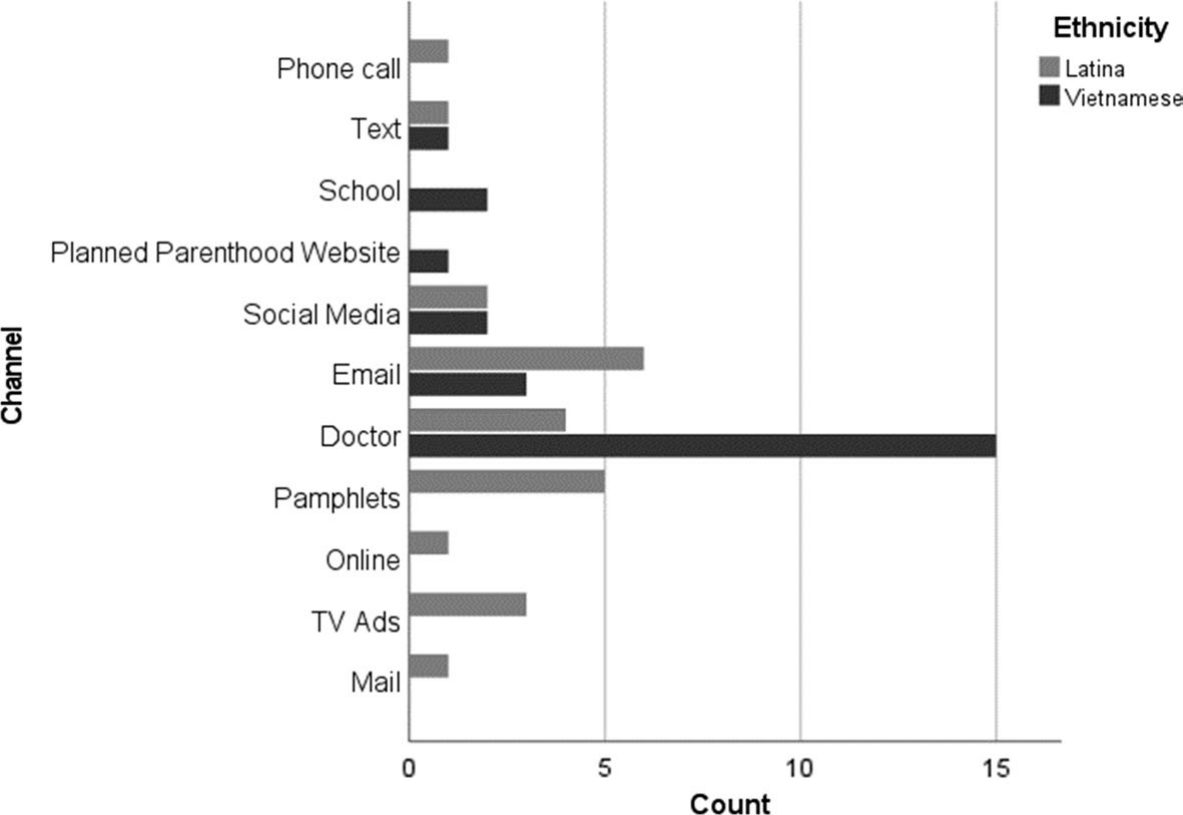
Frequency Distribution of Top Communication Preference by Ethnicity. Participants identified “school” as a preferred channel of communication to learn about the HPV vaccine, but they varied on their preferred school context (e.g., middle school, high school, or college)

The development of the interview guide was informed by CCT and explored women’s health information seeking generally (i.e., its sources) and preferences for receiving education about HPV vaccination, with follow-up probes exploring women’s motivations for turning to various sources to seek health information (e.g., convenience, anonymity, tailored information, access to medical expertise) and how they initially learned about HPV vaccination. A separate question explored their specific preferences for receiving the HPV vaccine. Women were asked how they would like to receive information related to HPV from Planned Parenthood and if they had additional preferences for receiving health information outside of Planned Parenthood (see Fig. 1). A separate interview guide was developed for interviewing the two staff members (one Vietnamese American and one White, both clinic operations managers at the two health centers) about how Planned Parenthood communicates with patients about HPV vaccine information.

The interview guide for the 30-min interview included eliciting preferences and information about where women get their health information (in addition to their medical providers) and their preferences for receiving HPV vaccine information in the future. To explore where women turn for health information and their motivations and preferences, we asked: “Where do you go to find health information?” and “Do you use your phone to seek health information?” We also asked women about their preferences for receiving HPV vaccine information: “We would like to hear your thoughts on how best to reach women like yourself (and men) about HPV vaccination.” Twelve demographic questions were asked at the end of the interview. During interviews with the two Planned Parenthood staff members, we asked about clinic visit protocols related to HPV vaccination recommendations and how educational material was provided to patients.

### Data Analysis

We transcribed the interview audio files verbatim for accuracy and replaced personal identifiers with pseudonyms. The data were imported into the qualitative data analysis software NVivo Pro 11. We took a phronetic iterative approach to our data analyses to code the data (Tracy, 2018, 2019b). Phronetic refers to the Greek term phronesis, which prioritizes understanding contextual knowledge. An iterative approach involved inductively analyzing the data for emerging themes and then using Channel Complementarity Theory (CCT) to deductively analyze women’s use and motivations of multiple channels for health information. We initially read all transcripts (data immersion as the first step in the data analysis process) of women’s descriptions of where they turned for health information, when and how they used each of these sources (i.e., what motivated their use), where they learned about HPV vaccination, and how they would prefer to receive future HPV vaccine information.

As an inductive approach, we conducted descriptive, line-by-line coding, tagging segments of the transcript data based on women’s descriptions of the various channels they turned to for general health information, motivations related to when and how women used those channels, followed by coding for preferences for receiving HPV vaccine information (Tracy, 2019a). We then developed a codebook organizing these primary descriptive codes by grouping them into themes related to types of sources women turn to, trusted sources, and characteristics that motivated use of specific channels. Organization of the codes into higher-order interpretive themes was guided by CCT (Dutta-Bergman, 2004b; Rains & Ruppel, 2016). We reviewed, jointly discussed, and resolved any discrepancies surrounding the organization and labeling of emergent interpretive themes (Morse, 2015; Thorne, 2020). In the last stages of data analyses, we compared and contrasted emergent themes: first within groups, i.e., each of the two racial and ethnic groups (Latina and Vietnamese); and then across groups in aggregate (Strauss & Corbin, 1998; Tracy, 2019a). Finally, we organized emergent themes based on how participants answered our questions; namely, the extent, context, and motivations of Latina and Vietnamese women concerning the sources to which they turned for health information and why (Tracy, 2018).

## Results

Information related to the respondents’ sociodemographic characteristics is reported in Table 1. Of the vaccinated participants, a majority (72%) completed the HPV vaccine series (three shots); Latina women completed the series to a lesser extent (50%) than did Vietnamese women (94%). Most participants (90%) reported being sexually active (83% Latina, 96% Vietnamese), whereas half or less reported their partners used condoms (46% Latina, 50% Vietnamese). Only about half of participants reported having ever received a Papanicolaou (Pap) test (50% Latina, 54% Vietnamese). Differences in marital status and having children were observed, with more Latina women reporting being married (13% compared with 0% Vietnamese), and having children (42% compared with 0% Vietnamese). Also, a greater proportion of Latina women (13%) reported having received a positive HPV test, compared to Vietnamese women (4%).

**Table 1:** Participant demographic characteristics

Variable	Unvaccinated Latina	Vaccinated Latina	Total Latina^a^	Unvaccinated Vietnamese	Vaccinated Vietnamese	Total Vietnamese^a^	Total Sample^b^
	(*n* = 8)	(*n* = 16)	(*n* = 24) *n* (%)	(*n* = 8)	(*n* = 16)	(*n* = 24) *n* (%)	(*N* = 48) *n* (%)
Age in years (Mean ± *SD*)	23 ± 1.9	23 ± 2.5	23 ± 2.29	21 ± 2.4	22 ± 2.5	22 ± 4.95	22 ± 2.4
HPV vaccination status							
1 or 2 shots	0	8	8 (50)	0	1	1 (6)	32 (66)
Completed series	0	8	8 (50)	0	15	15 (94)	23 (48)
Currently sexually active	7	13	20 (83)	8	15	23 (96)	43 (90)
Condom use^c^	4	7	11 (46)	5	7	12 (50)	23 (48)
Has health insurance	6	13	19 (79)	6	12	18 (75)	37 (77)
Ever received a Pap test	3	9	12 (50)	3	10	13 (54)	25 (52)
Partner status							
Married	1	2	3 (13)	0	0	0 (0)	3 (6)
Dating or engaged	6	7	13 (54)	7	15	22 (92)	35 (73)
Single	1	7	8 (33)	1	1	2 (8)	10 (21)
Has children	3	7	10 (42)	0	0	0 (0)	10 (21)
HPV exposure^d^	0	3	3 (13)	0	1	1 (4)	4 (8)

^a^Percentages displayed out of total racial and ethnic sample (*n* = 24); however, for vaccination status, the denominator is 16, not 24, because 16 women were vaccinated

^b^Percentages displayed out of total sample (*N*= 48)

^c^Participants were asked, “Do you currently use condoms and/or dental dams?” The interviewer then asked the participant to clarify which method they use if not specified in the original response

^d^Participants were asked to indicate their history exposure, with response categories of: (a) positive HPV history, (b) negative HPV history, and (c) never been tested

### Latina and Vietnamese American Women Use Many Sources for General Health Information

Overall, women described using a range of online sources to seek general health information, such as the internet, social media platforms, email, and mobile or cell phone texting. They also reported turning to in-person medical providers or family members for health questions and concerns. Latina and Vietnamese women’s top communication source preferences are reported in Fig. 1. Vietnamese women valued in-person visits with medical doctors to a greater degree than other sources for health information, whereas Latina women described a wide range of preferences for health information sources.

### Immediacy and Accessibility of Information, Convenience, and Credibility Are Critical

Although in-person medical consultations were described as ideal, the immediacy, accessibility, and convenience of online health information outweighed other sources due to time constraints and scheduling barriers. We received feedback from vaccinated and unvaccinated participants. Lourdes, an unvaccinated 23-year-old Mexican woman, expressed her lifestyle’s impact on health information seeking:

For me, I’m always on the go. … Sometimes I log online or I’m on Facebook. I mostly always honestly review all the news because it’s all there. I feel like online would be an appropriate source, because I don’t watch much TV.

On the other hand, a vaccinated 20-year-old Vietnamese woman, Katrina, said:

I think I prefer the doctor because it’s accurate that way, but out of convenience, I would prefer the Internet and then I would research more to get more information. … I’ll go on a credible website and find out more.

Mindful of trustworthiness, women expressed a preference for easily accessible, convenient, and immediate online information.

Women also described the importance of seeking credible information with a heightened awareness of its value. For example, Carmen, an unvaccinated 24-year-old Mexican woman, mentioned:

I try not to just go to any website. … I try to look up some things and also seek medical advice from a doctor or from a nurse, from someone that is trained in that. I think that is the best way.

Megan, a vaccinated 25-year-old Vietnamese woman, described using.org or.gov sites: “I ask my provider for resources.”

### For Receiving HPV Vaccine Information, Interpersonal Engagement, Trusted Sources, and Convenience Are Important

When asked how they would prefer to receive HPV vaccine information, women attending Planned Parenthood health centers described a variety of sources: the Planned Parenthood website, phone text messages, social media, and in-person medical visits. For many women, an in-person visit with a doctor was important, ideal, and preferred for learning more about HPV vaccination. Tania, a vaccinated 26-year-old Mexican woman, said: “I accept brochures and everything, but if they have information to give me, I mostly prefer through conversation.”

Similarly, Linda, a vaccinated 24-year-old Vietnamese woman, shared:

The doctor is more reliable, you know? But I’m more of a visual learner, so I would like to read the information. If someone was talking to me for 10, 15 mins about a long subject, I’m just like, “Whoa, I didn’t get all that. What was that?”

Carrie, a Planned Parenthood staff member serving primarily Latina women, described in-person counseling as the “most effective,” but also the most difficult due to limited time with the patient. She described the need for other communication approaches that can inform patients of additional HPV information, but other forms of communication come with challenges. “Handouts I think are very effective, but then of course, I see lots of handouts [discarded] in the parking lot.” Although Carrie described handouts as a useful resource for the population she serves, abandoned handouts may indicate they are not the most desired platform for all patients. Last, Christina, a Planned Parenthood staff member serving a high number of Vietnamese patients, also stated “face-to-face is best” for communicating HPV information. She later described, “Patients zone me out. [They say] ‘I’m here for birth control, I don’t need anything else.’ … If they’re not interested, let me at least slide the HPV sheet into your bag.” Christina described how a highly effective source is not always the most convenient for every patient and emphasized the need to provide health information through multiple sources. “I’m going to say to be honest with you, maybe a combination of multiple [sources] would be good.”

Women also discussed the importance of receiving HPV vaccine information from trusted sources. As part of their vaccine decision stories, women talked about learning or wanting to receive HPV vaccine information from four sources: Planned Parenthood, school, doctors, or mothers. They described health information from these sources as trusted health information. Elizabeth, a vaccinated 20-year-old Vietnamese woman, stated, “I know that Planned Parenthood sends out text messages and I pay attention to those.” A vaccinated 21-year-old Vietnamese woman, Vivi, expressed her tendency to receive information through her school or the doctor because of her fear of misinformation on the internet. She said, “I typically [receive] health information from school since I’m going into the medical field, but sometimes going to the doctor will also help me be aware of diseases. … I’m too scared to Google things.” Similarly, Lourdes, a vaccinated 19-year-old Mexican woman, elaborated: “My mom works for the county, so she’s always sending me health information. That’s how I gain most of my health information, from my mom. Because she is very aware of all that stuff.” Interestingly, peers were not cited as trusted sources, with more women describing clinicians, health clinics, schools, and mothers as trusted sources for health information.

Convenience seemed to reflect women’s preferences to receive HPV vaccine information. Many women discussed receiving messages through social media, text, or email, given their daily use of these platforms. The sheer convenience, ubiquity, and pervasiveness in daily life of these technologies seemed to dictate their use as sources for health information in this age group. Consuelo, an unvaccinated 23-year-old Mexican participant, shared:

I feel like pamphlets get thrown away a lot and no one really looks at it. … I think emails are good because you’re looking through all the messages you have gotten and probably read it more. … Social media I think is good because people are on that all day. Maybe they’ll see something to make them think of doing it [vaccinate]. Text messages are also good. People are like, “What is this number?’ They will read it and so that will be good, too.

A vaccinated 22-year-old Vietnamese woman, Annie, described exposure through Facebook: “I mean, everyone’s on it. And then I only see little articles, which I’ll open up and read. I think Facebook would be something good if you could get someone’s attention that way.” Women’s preference for social media channels appeared to reflect their routine use of this technology. Vy, an unvaccinated 22-year-old Vietnamese participant, conveyed her preference for receiving health information through email:

I think I would prefer email because I check it more. I probably wouldn’t want a text just because it’s a little more personal and a little more annoying … but with email, I check it once a day or once every couple of days.

### Avoiding Information Overload and Protecting Privacy in HPV Vaccination Interventions

Despite the ease and availability of certain sources, Latina and Vietnamese women voiced concerns about receiving too many messages by text (message fatigue) and receiving sexual health texts that family members might see (privacy concerns). Message fatigue, particularly if delivering health interventions, via mobile phones should be carefully considered given the competitive message environment. Women expressed having experienced text message fatigue and cautioned about delivering an HPV vaccine intervention via many emails or texts. Information overload is another consideration for dissemination of health interventions. Diana, a vaccinated 21-year-old Mexican American woman, shared:

It depends on how many [emails or text] you are sending. If you are sending one here and there, I think that would be pretty good, but if it’s over and over, women wouldn’t want to keep on looking at them or they are going to say, “Oh, it’s just the same thing.”

Participants expressed that too many text messages would deter them from opting into a system to receive information. For example, Elizabeth, a vaccinated 20-year-old Vietnamese woman, stated: “If it’s too frequent, it gets annoying. … I would unsubscribe.”

In this study, women also described the importance of privacy, especially in the context of receiving HPV vaccine messages and the possibility of family members reading or seeing their HPV vaccine messages on their mobile phone. A vaccinated 26-year-old Mexican American woman explained that she had received the first HPV vaccine in the emergency room at the age of 21, but her mother found out about it through a telephone call from the hospital and became upset.

I remember they gave my mom a call for my second shot. She was kind of mad that I got it (the HPV vaccine). But I told her they just told me to get it and I did. … She probably thought I was getting it because I was sexually active.

Her mother feared her daughter may be receiving this shot because she is sexually active before marriage, a behavior that conflicts with cultural and religious beliefs. This woman portrayed sentiments similar to other women who feared their mother’s response to requesting HPV vaccination, which highlights the need for communication sources that offer privacy for young adults.

Among Vietnamese women, privacy concerns manifested similarly. A vaccinated 19-year-old Vietnamese American woman, Kathy, said:

With me, I don’t want my parents to see, so text message would be really inconvenient for me because if there’s a situation where my mom just glances at my phone and there’s a pop-up saying “Sexual health blah blah,” I would be in deep shit. … It would be really inconvenient because I have parents who may look over my shoulder or check my phone to see the time and they’ll see a notification.

Particularly for sexual health, text message notifications were described as problematic due to disclosure fears to others, particularly parents.

## Discussion

Young Latina and Vietnamese American women visiting Planned Parenthood health centers in Southern California described accessing multiple sources for general health information that ranged from Google, YouTube, and social media to in-person Planned Parenthood staff, doctors, mothers, and school health classes. For seeking general health information, communication channel characteristics that motivated women’s use included immediacy, access, convenience, and credibility. Because we were interested in having results inform the dissemination of an HPV vaccine EBCCP, we also asked women to share their communication preferences for receiving HPV vaccine information. In this context, women valued interpersonal engagement, privacy, avoiding information overload, and using trusted sources to deliver the message, which included Planned Parenthood staff, mothers, school health classes, and doctors.

Our results have implications for adapting the dissemination of a video-based HPV vaccine EBCCP. Latina or Vietnamese women did not prefer any one particular source, which would have suggested the need for a singular delivery modality for intervention dissemination. Searching for and receiving health information from diverse sources appears to be the new norm, as reflected during the coronavirus pandemic and in a study of men’s source preferences for HPV vaccine information (Ali et al., 2020; Pew Research Center, 2019; Sledge et al., 2019; Vogels, 2019). Young Latina and Vietnamese women described searching the Planned Parenthood website for sexual health information, but they also searched various social media sites including Facebook, Snapchat, Instagram, and YouTube.

Findings regarding where women turn online are similar to reported trends among younger adults across the United States, many of whom visit social media sites daily in parallel with increasing access to health information (Smith & Anderson, 2018). Social media channels including YouTube, Facebook, and Instagram represent sources that are accessed and checked daily, especially by young adults. An emergence of digital interpersonal networks to coordinate daily life is normative in this age group and could be used to share health prevention messages (e.g., WhatsApp or Facebook messenger group chats; Duong & Hopfer, 2021).

Latina and Vietnamese participants’ descriptions of multiple online sources were qualified insofar as they expressed that they valued in-person clinician interactions. For receiving HPV vaccine information, Latina and Vietnamese women valued interpersonal trusted sources and convenience. CCT recognizes individuals’ joint use of many sources, including interpersonal medical, family, and friend networks as a complement to online networks, rather than displacing one source for another (Rains & Ruppel, 2016). Receiving HPV vaccine recommendations across multiple sources reinforced the importance of vaccinations for women.

Participants’ use of various online and social media sources reflected their “on the go” lifestyles and time management needs—namely, the importance of source characteristics of convenience and immediacy. Online sources often serve the instant gratification and immediacy need that characterizes contemporary health-seeking behaviors as specified by Uses and Gratification Theory. This theory takes an audience centered approach asserting that people use media to gratify specific wants and needs (Katz et al., 1973; Whiting & Williams, 2013). Valuing convenience reflects the modern era, in which the most accessible source often determines which health messages receive the most exposure.

Many women expressed their preference to receive HPV vaccine recommendations from in-person medical consultations but, in their actual health information seeking, they relied on a range of sources. Individuals, especially young adult minority women, have become more sophisticated in their health information seeking, and have a heightened awareness of the importance of credibility (Moran et al., 2016). Health communication strategies in the digital age need to incorporate credibility cues that permit access to medical expertise online (Hu, 2015; Ruppel & Rains, 2012).

Our findings, especially regarding the channel characteristics that motivate young women’s use of different sources about HPV vaccination and, ultimately, drive their exposure to prevention messages, contribute to the prevention and dissemination science literature (Sloboda et al., 2014). Access, convenience, and immediacy to health information sources appear to drive use and play an important role in addressing health disparities by reaching medically underserved populations. Similar to research by Ruppel and Rains (2012; also see Rains & Ruppel, 2016), we found that women’s preferences and motivations for using certain channels for health information were driven by convenience and anonymity when seeking information online. Identifying source characteristics that are important for seeking and receiving health information is increasingly critical to effectively reach underserved populations with prevention messages as the availability of and access to online health sources become more fragmented (Din et al., 2019). Investigating online health sources that Latina and Vietnamese women turn to and the characteristics that drive their use of certain channels is important to disseminating and adapting prevention interventions because the choice of information sources is expanding, access to sources may differ depending on group, and source use may differ by age and race and ethnicity.

Women were mindful of evaluating the credibility of a source. Women’s preferences were influenced by whether they trusted the source, describing health care practitioners, the PPOSBC website, schools, and mothers as the most trusted for HPV vaccine messages. Consistent with past research, trust has been identified as an increasingly important consideration that contributes to whether audiences accept health messages (Hesse et al., 2005; Westerman et al., 2014). Health educators should therefore consider using one of the four trusted sources described by Latina and Vietnamese women to reach them: Planned Parenthood staff, doctors, mothers, or school-based messaging.

Similar to research involving men (Sledge et al., 2019), women expressed a preference for receiving information about HPV vaccination in person from their medical providers. Particularly for vaccine recommendations, studies have shown a preference for interpersonal medical interactions with access to medical experts as a key source characteristic (Hopfer & Clippard, 2011; Rains & Ruppel, 2016). The preference for interpersonal interactions with medical professionals underscores the important role clinicians play in communicating HPV prevention recommendations to young adults and disseminating interventions (Sloan & Knowles, 2017). However, the use of other sources highlights the significant role of accessibility and immediacy in exposure to health messages.

To effectively reach young Latina and Vietnamese women with HPV vaccine prevention messages, source preferences should be considered (Hopfer, et al., 2017a, 2017b). Findings suggest that the use of many sources by Latina and Vietnamese women should inform dissemination strategies for the NCI EBCCP HPV Vaccine Decision Narratives. During dissemination of the intervention, multiple sources should be employed if possible. This finding might suggest an integrated marketing communication approach to adapting the EBCCP to reach women across many platforms in a coordinated and integrated way, ideally with the same trusted message (Evans & Hastings, 2011; Kitchen et al., 2004). Such an approach not only repeats the same messages through multiple sources but also emphasizes creating a unified and seamless message for the audience—in this case, minority women.

Additionally, disseminating the EBCCP from perceived trusted sources such as Planned Parenthood, schools, and clinicians or circulated from parent organizations (e.g., mommy blogs) should be considered. State public health agencies disseminate statewide campaigns using many sources (Cartmell et al., 2018). A multisource approach increases the probability of exposure and accommodates the complex manner in which women use or disregard health information. Using multiple sources reinforces messaging that can robustly affect behavior change (Snyder & Hamilton, 2002).

An alternative or complementary dissemination strategy might include having women self-select their channel preference when visiting Planned Parenthood health centers. Having the target audience self-select its preference for receiving health information after a clinic visit has been successfully employed in a previous vaccine study (Kempe et al., 2016). This strategy also coincides with PPOSBC’s switch to streamlining its educational material online and inviting women to choose which prevention material they wish to receive as part of their women’s health portal (N. Ramirez, personal communication, October 26, 2018). Target audiences are more receptive and satisfied when they have input regarding how they receive health information. Tailoring education by patient preference has been recognized as a key communication strategy to successfully deliver health promotion materials and change behaviors (Kreps, 2017). Including material that is audio based (e.g., videos) might make educational material more accessible to immigrant populations with low literacy skills.

The women in our study evaluated the trustworthiness of sources, giving more weight to familiar and credible health care organizations such as PPOSBC. Concurrently, many women discussed the accessibility of mobile and social media messages. The combination of strategies demonstrates the importance of connecting target audiences with trusted networks in the digital age (Kreps, 2017). Although much discussion in the literature on tailoring has been focused on content, our study’s findings suggest consideration of tailoring intervention dissemination by source preference and the attributes that motivate its use (e.g., accessibility, immediacy).

Being mindful of privacy concerns when disseminating HPV vaccine messages via phones is also important. Designing interventions that minimize disclosing sensitive topics inadvertently to family or significant others is imperative to protecting patient privacy. Another concern involved sending too many messages, which can lead to disregarding messages due to fatigue (Kim & So, 2018). Given the source characteristics and concerns women described, health intervention researchers will need to accommodate the changing landscape of media consumption patterns and consider information overload (Michie et al., 2017).

### Limitations

This study was conducted in a single U.S. geographical area, reflecting the source preferences of Latina and Vietnamese American women in Southern California. Additional sources and source characteristics may be uncovered when sampling Latina and Vietnamese women in other geographic areas. Our study aims focused on identifying the range of sources these women turned to and understanding what characteristics drive those choices. Although social desirability bias may have affected responses, we sampled both vaccinated and unvaccinated women to uncover a range of responses. Social desirability is problematic when it results in diminished heterogeneity in responses. We implemented a number of techniques to minimize social desirability: we trained interviewers to open the conversation by emphasizing that there are no right or wrong answers, building rapport, and explaining the purpose of the study to inform designing an intervention. Finally, we integrated into the interview guide follow-up questions or prompts to explore vague responses (Bergen & Labonte, 2020). Claims about whether differences between Latina and Vietnamese women exist in the sources they use for health are beyond the scope of this study. Rather, these findings highlight key communication sources, channels, and the characteristics that motivate their use among Latina and Vietnamese women.

### Conclusion

The growth, reach, and range of communication technologies continue to expand and change rapidly in the digital environment, shaping the way women and families seek and receive health information. Social media is increasingly used to access health information, especially among young adult minority women, to complement in-person medical care (Smailhodzic et al., 2016). This study adds to the sparse literature specific to Vietnamese and Mexican American minority subgroups. Attending to communication channel characteristics and a target group’s motivation to use multiple channels is now a necessary adaptation step in intervention dissemination planning and prevention science. Self-tailoring prevention interventions by source preference or applying an integrated communication marketing approach will likely be key to effectively reaching young Latina and Vietnamese American women who are disproportionately affected by HPV-associated cancers with important prevention messages.
